# On the Mechanisms of the Effects of Ionizing Radiation on Diblock and Random Copolymers of Poly(Lactic Acid) and Poly(Trimethylene Carbonate)

**DOI:** 10.3390/polym10060672

**Published:** 2018-06-16

**Authors:** Agnieszka Adamus-Wlodarczyk, Radoslaw A. Wach, Piotr Ulanski, Janusz M. Rosiak, Marta Socka, Zois Tsinas, Mohamad Al-Sheikhly

**Affiliations:** 1 Institute of Applied Radiation Chemistry, Lodz University of Technology, Wroblewskiego 15, 93-590 Lodz, Poland; agnieszka.adamus@p.lodz.pl (A.A.-W.); piotr.ulanski@p.lodz.pl (P.U.); rosiakjm@mitr.p.lodz.pl (J.M.R.); 2 Department of Polymer Chemistry, Centre of Molecular and Macromolecular Studies, Polish Academy of Sciences, Sienkiewicza 112, 90-363 Lodz, Poland; msocka@cbmm.lodz.pl; 3 Department of Materials Science and Engineering, University of Maryland, 4418 Stadium Dr., College Park, MD 20742-2115, USA; ztsinas@umd.edu (Z.T.); mohamad@umd.edu (M.A.-S.)

**Keywords:** electron beam irradiation, crosslinking, scission, PLA, PTMC, poly(trimethylene carbonate-*co*-d-lactide), diblock copolymers, random copolymers, EPR, alkoxyl radicals, acetyl radicals

## Abstract

This article demonstrates that ionizing radiation induces simultaneous crosslinking and scission in poly(trimethylene carbonate-*co*-d-lactide) diblock and random copolymers. Copolymer films were electron-beam (EB) irradiated up to 300 kGy under anaerobic conditions and subsequently examined by evaluation of their structure (FT-IR, NMR), molecular weight, intrinsic viscosities, and thermal properties. Radiation chemistry of the copolymers is strongly influenced by the content of ester linkages of the lactide component. At low lactide content, crosslinking reaction is the dominant one; however, as the lactide ratio increases, the ester linkages scission becomes more competent and exceeds the crosslinking. Electron paramagnetic resonance (EPR) measurements indicate that higher content of amorphous carbonate units in copolymers leads to a reduction in free radical yield and faster radical decay as compared to lactide-rich compositions. The domination of scission of ester bonds was confirmed by identifying the radiolytically produced alkoxyl and acetyl radicals, the latter being more stable due to its conjugated structure.

## 1. Introduction

Biodegradable polymers are excellent solutions for a wide range of applications, including those in biomedical and pharmaceutical fields. Among medical polymers, aliphatic polyesters of lactides and poly(trimethylene carbonate) (PTMC) have been extensively investigated due to their favorable properties, such as toxicological safety, controlled biodegradability and, if blended or copolymerized, tailorable mechanical properties [1,2,3].

Poly(lactic acid) (PLA) is a well-known typical biodegradable and biocompatible polymer, which is commonly produced from renewable resources [4]. This rigid polymer is utilized in load-bearing implantable medical devices and drug release matrices. It is investigated towards applications as disposable primary commodities or environmentally friendly packaging materials [5,6]. Biodegradation of lactides occurs through hydrolysis of ester linkages in the main chain, yielding naturally occurring lactic acid. Nevertheless, its poor thermal stability and deficient mechanical properties (high stiffness) limit the area of PLA application [7,8]. To improve heat stability and mechanical properties, various methods such as annealing, adding nucleating agents, fillers in form of fibers or nanoparticles were applied [9,10,11,12]. Alternatively, specific chemical and physical treatments were also applied to introduce crosslinking between PLA macromolecules to alter disadvantageous properties [13,14,15].

Opposite to PLA, PTMC is a flexible, biodegradable, and biocompatible synthetic polymer, showing attractive surface erosion behavior in vivo. Compared to the relatively rigid polyesters based on glycolide or lactides, materials with lower modulus might have advantageous properties for medical applications in soft tissue engineering or drug delivery systems [16,17,18]. However, PTMC has not been widely used in medical applications, mainly because its unsatisfactory low tensile strength and creeping tendency that significantly limits its application for other uses. For this reason, TMC monomer is often used in copolymers with different lactones, for instance to manufacture surgical sutures, e.g., lactide [19], ε-caprolatone [20], glycolide [21].

Combination of the two components of different properties, the lactide and PTMC, results in a material more flexible than pure PLA, but with much improved mechanical properties as compared to PTMC. Therefore, introducing PTMC into PLA through copolymerization, i.e., synthesis of poly(lactic acid)-*co*-poly(trimethylene carbonate), becomes an important way to fine-tune important properties of PLA. Such copolymers may be utilized in variety of current and emerging applications, e.g., resorbable sutures, long or short term implantable devices, microparticles for drug delivery systems, bone graft substitutes, etc. [22,23,24,25].

Response of the organism to the implanted biodegradable material, besides its assumed biocompatibility, depends on numerous factors, such as place of implantation and the surrounding tissue, chemistry of the material, mechanical, morphological, and surface properties of the implant, mechanism, and kinetics of biodegradation along with metabolism or resorption of degradation products. One of the important issues in the manufacturing of medical devices is providing sterility. Several procedures can be employed for sterilization of PLA or PTMC polymers, such as sterilization with ethylene oxide gas [26], low-temperature plasma, injection molding process, steam [27,28]. On the other hand, radiation processing and the commercially dominating sterilization process by high-energy radiation have been reported for modification and sterilization of PLA [29,30,31] and poly(d-lactic acid) (PDLA)-based composite materials [32], yet with severe restrictions. Because of high efficiency, excellent penetration characteristics and financial benefits of using ionizing radiation, radiation sterilization technique is widely applicable and eliminates problems which may appear with other sterilization methods, such as for instance high temperature, penetration of ethylene oxide gas or hydrogen peroxide (for cold plasma sterilization method), toxic residuals and need for quarantine period, surface chemistry change, or non-uniformity of sterilization.

Nevertheless, radiation sterilization of polymers may also be problematic due to the damaging effects of radiation on some polymeric materials. Radiation can modify the physical and chemical properties of polymers through main-chain scission and crosslinking. Upon irradiation, free radicals are formed randomly along polymer chains. They can react with each other or initiate other reactions, mainly chain scission (degradation). This in turn gives rise to changes in chemistry and material properties. A recombination of two macroradicals leads to crosslinking, which generally results in enhancement of physical properties, whereas degradation, manifesting itself by reduction in molecular weight, typically leads to detrimental changes of mechanical properties. In many polymers both processes take place simultaneously and the final results of irradiation depend on the ratio of their efficiencies, which can be quantified as radiation-chemical yields of intermolecular crosslinking and chain scission, thus chemical changes and yields should be always determined prior to further physical properties testing [33,34].

The behavior of both PLA and PTMC homopolymers under irradiation is relatively well known. Poly(lactic acid) has high mechanical strength, but in the pure form, without additives, it cannot be effectively modified or sterilized using ionizing radiation because it degrades readily while irradiated, leading subsequently to a polymer of lower molecular weight and lesser properties [35]. There are some reports concerning radiation-induced crosslinking of PLA using an added crosslinking agent, for instance triallyl isocyanurate, but the additive is biologically unsafe [35,36]. PTMC undergoes simultaneous degradation and cross-linking initiated by radiation, nevertheless the latter process predominates, which results in an increase in the molecular weight. Therefore, PTMC can be sterilized by radiation technique [37].

Although some data on the effects of electron-beam (EB) irradiation on PLA and its copolymers have been published [38,39,40], no studies have been reported so far regarding the effects of radiation on the properties of PLA-*co*-PTMC copolymers. In the present work, chemical changes occurring upon irradiation of diblock and random copolymers of various molar compositions were investigated. Molecular weight, intrinsic viscosity and thermal properties were examined as a function of electron beam irradiation. Subsequently, radiation chemical yields of chain scission and crosslinking in copolymers were evaluated, thus copolymers of compositions and microstructure that are prone to scission or crosslinking were identified. Yield and decay of radicals formed in irradiated systems were examined as a function of copolymer molecular structure by electron paramagnetic resonance spectroscopy (EPR).

Ionizing radiation induces several reactions in polymers, of which those leading to main chain scission and to crosslinking of macromolecules are the most important from the point of view of polymer physical properties and of its suitability for particular applications. Scission of only a few bonds in the polymer backbone may cause tremendous alternation of mechanical properties due to reduction of polymer molecular weight. On the contrary, intermolecular crosslinking results in an increase of molecular weight of the polymer, therefore one may expect improvement of physical properties of the material. In some cases, when crosslinking predominates over degradation, a chemical (covalent) gel may be formed and the polymer becomes insoluble. It is anticipated that modification of physical and chemical properties may be accompanied by only minor changes in chemical composition of the polymers.

## 2. Materials and Methods

### 2.1. Materials and Polymers Synthesis

Homopolymers of PTMC and PDLA were used as reference in physicochemical characterization. Commercially available poly(-lactic acid) (Dow-Cargill PLLA, Minneapolis, MN, USA, *M*_n_ of 79 kg·mol^−1^) was used without further purification. For PTMC synthesis polymer-grade 1,3-trimethylene carbonate (TMC) (Boehringer Ingelheim, Ingelheim am Rhein, Germany) and stannous octoate (SnOct_2_) (stannous 2-ethylhexanoate) (Sigma, San Jose, CA, USA) were used as received. Poly(trimethylene carbonate) (PTMC) was synthesized in dried, freshly silanized (Serva solution, Boehringer Ingelheim, Ingelheim am Rhein, Germany) glass ampoules. The ampoules were purged with dry argon, charged with monomer and catalyst (2 × 10^−4^ mol SnOct_2_ per mol TMC), and heat-sealed under vacuum. The polymerizations were conducted at 130 ± 1 °C for 3 d. The polymers were purified by precipitating their chloroform solutions into methanol and then dried [2].

For synthesis of copolymers lactide and carbonate were used. Lactide monomer of DLA (Boehringer Ingelheim, Ingelheim am Rhein, Germany) was crystallized from dry 2-propanol and then purified by sublimation in vacuum (10^−3^ mbar, 90 °C). TMC (Boehringer Ingelheim, Ingelheim am Rhein, Germany, >99%) was crystallized from dry tetrahydrofuran/ethyl ether mixture (3/1) and sublimed (10^−3^ mbar, 45 °C). THF (Sigma-Aldrich, Darmstadt, Germany) solvent was purified as described previously [41]. Aluminum tris-isopropoxide (Al(OiPr)_3_) used in a form of its trimer ({Al[OCH(CH_3_)_2_]_3_}_3_ was prepared from the commercial alkoxide (Sigma-Aldrich, Saint Louis, MO, USA, 98%) as described elsewhere [42]. Bidendate ligand, (R)-(−)-2,2′-[1,1′-binaphtyl-2,2′-diylbis(nitrylomethilidyne)] diphenolate (SB(OH)_2_), was prepared as described in ref. [43].

Synthesis of copolymers followed the procedure given below [44]. Reacting mixtures were prepared in sealed glass vessels using a standard high vacuum technique. Breakseals equipped with glass hammers, which separately contained Al(OiPr)_3_ and monomers, were sealed in the reaction glass vessel. Tetrahydrofuran (THF) was distilled into this vessel under vacuum. The SBO_2_Al-OiPr initiator is formed in situ from the equimolar quantities of SB(OH)_2_ and Al(OiPr)_3_, kept for 24 h in THF as solvent at 80 °C just before use. The breakseals that contained monomers then were broken and all components were mixed at room temperature. The resulting reaction mixture was distributed into a several glass vials, and placed into a thermostat at 80 °C. The random copolymers of DLA and TMC were obtained via simultaneous ring-opening polymerization (ROP) initiated with Al(OiPr)_3_, with segmental exchange side reaction. The diblock copolymers were synthetized via sequential ROP in the presence of (R)-SBO_2_Al-OiPr, proceeding with suppression of segmental exchange. The resulting copolymers were precipitated into cold methanol, separated, and dried in vacuum at room temperature to a constant weight.

### 2.2. Preparation of Samples and Irradiation

The copolymers were dissolved in chloroform at 20 wt % and solutions were drop-casted to form films of ca. 50 μm thickness. The films were dried to constant weight at room temperature under vacuum. The film samples were vacuum-sealed in plastic bags making barrier to limit air contact and irradiated by electron beam (6 MeV linear accelerator) at room temperature. The dose rate was 5 kGy·min^−1^ (as determined by alanine dosimetry) and the applied absorbed doses were up to 300 kGy. Alternatively, for EPR investigations, the copolymers were irradiated with an electron beam (7 MeV linear accelerator) to 30 kGy at the dose rate of 10 kGy·min^−1^ (determined by alanine dosimetry) in plastic pouches without air access at dry-ice temperature and transferred to EPR quartz capillary immediately after irradiation under neutral gas atmosphere (Ar).

### 2.3. Analytical Procedures

Infrared spectra were obtained on an Nicolet Avatar TM 330 FT-IR Spectrometer (Thermo Electron Corporation, Waltham, MA, USA) in the HATR mode. Approximately 0.4 ml of 0.1% polymer solution in chloroform was casted onto the IR transmitting window (80 mm × 10 mm ZnSe plate) to form a uniform layer. The plates were dried for 24 h to remove traces of solvent before spectra acquisition. Spectra were collected with a resolution of 4 cm^−1^ and with 64 scans per sample over the range of 4000–500 cm^−1^.

The polymer samples were analyzed by proton nuclear magnetic resonance (^1^H-NMR) spectroscopy using a 500 MHz Bruker spectrometer (Billerica, MA, USA) with dichloromethane-d2 as the solvent.

Intrinsic viscosities ([η]) of copolymers were determined in chloroform at 25.0 °C with an AVS-310 automatic viscometry system (Schott Geräte, Mainz, Germany) equipped with a 01/0a type Ubbelohde viscometer (Schott Geräte, Mainz, Germany). Prior to the analysis, the non-irradiated samples were filtered through 0.45 μm pore size filters (Sartorius), while the irradiated samples were analyzed without prior filtration.

Gel permeation chromatography (GPC) measurements were conducted using the system equipped with a P580 pump (Dionex, Sunnyvale, CA, USA), two columns of 10 μm and 5 μm pore size (Knauer, Biberach/Baden, Germany) and three detectors: Viscotec Ralls Detector (static light scattering at 90° at a wavelength of 670 nm) and Viscotec Dual Detector 250 (refractometer/viscometer, Houston, TX, USA). Dichloromethane was used as the eluent at 30 °C at a flow rate of 0.8 ml·min^−1^. Sample concentrations in the range 5–10 g/L and injection volumes of 100 μL were used. All solutions were filtered prior to injection into the GPC through 5 μm PTFE membrane filters (Sartorius).

Thermal properties of copolymers were determined by differential scanning calorimetry (Q200 M-DSC, TA Instruments, New Castle, DE, USA). Samples of ca. 5 mg sealed in aluminum pans were analyzed in the temperature range from −60 to 210 °C at a heating rate of 10 °C·min^−1^ under nitrogen atmosphere. The degree of crystallinity of lactide component (*χ*_c_) was calculated using the following formula:

(1) $$\chi_{c}=\frac{\Delta H}{\Delta H_{o}}\;\left(\mathrm{lactide}\mathrm{wt}\%\right)$$

where Δ*H* is enthalpy of fusion of examined sample, Δ*H*_o_ denotes the enthalpy of fusion of 100% crystalline polylactide sample of 93.6 J·g^−1^ [45]. Correction on cold crystallization was used whenever necessary.

EPR spectra of free radicals in poly(TMC-*co*-DLA) copolymers were collected at room temperature using an ESP300 spectrometer (Bruker Biospin, Billerica, MA, USA) with the following instrument parameters: microwave frequency of 9.42 GHz, microwave power 0.5 mW, frequency modulation 100 kHz, modulation amplitude 3.12 G, receiver gain 6.32 × 10^3^, center field at 3350 G, sweep width 500 G, conversion time 40.96 ms, and time constant 20.48 ms. It was verified that the modulation amplitude, as well as the ratio of the conversion time to time constant, did not distort the signal. The relative concentration of radicals was determined by double integration of signals recorded directly upon irradiation and after different time. The spin concentration in irradiated samples was determined following procedures described in [46].

## 3. Results and Discussion

### 3.1. Synthesis of Copolymers

A series of diblock and random copolymers of PTMC and PDLA was synthesized according to procedures described above (for details see Socka et al. [44]). The polymerization was monitored with NMR and GPC to evaluate the progress and the outcome of the synthesis. Targeted molecular composition is presented in Table 1 together with actual composition of the obtained copolymers, as determined by NMR, degree of monomer conversion and average molecular weights evaluated by GPC.

The molecular composition of the copolymers synthesized with high yield was in good agreement with the targeted one. The microstructure of polymers resulted from simultaneous polymerization of two monomers, in terms of average lengths of DLA and TMC microblocks revealed average, yet satisfactorily randomness [44]. Molecular weights of diblock copolymers were of standard level that can be obtained by sequential polymerization, therefore the anticipated molecular weights of random copolymers were at similar level.

### 3.2. Irradiation Effects on the Structure of Copolymers

**Figure 1:**
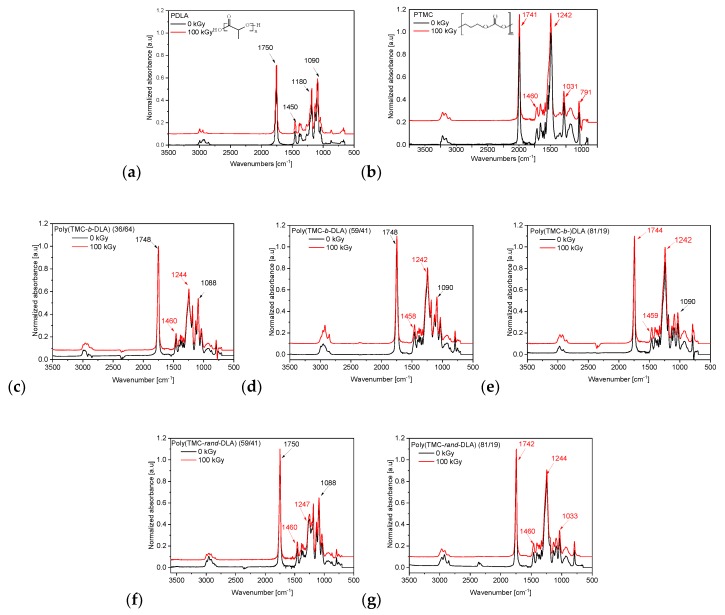
FT-IR spectra of PDLA (**a**), PTMC (**b**), poly(TMC-*b*-DLA) diblock copolymers of different PTMC content ((**c**)-36%, (**d**)-59%, (**e**)-81%) and poly(TMC-*rand*-DLA) random copolymers of different PTMC content ((**f**)-59%, (**g**)-81%) before irradiation (black and lower lines) and after irradiation at 100 kGy (red and upper lines).

To confirm the incorporation of the monomers in the synthesized copolymers, a series of FT-IR measurements were conducted. FT-IR spectra of PLA and PTMC homopolymers and their copolymers of various compositions are presented in Figure 1 (black lines). Spectra of PLA are characterized by bands at 1750 and 1180 cm^−1^ assigned to carbonyl and ether bonds, respectively [47]. Similarly, the carbonyl and ether bonds of PTMC are detected at 1741 and 1242 cm^−1^ [48]. In the spectra of the poly(TMC-*b*-DLA) copolymers the vibration of the ether bonds can be seen as two bands at 1184 and 1244 or 1242 cm^−1^ for copolymers containing 36% and 59% of PTMC, respectively. These bands correspond to those observed in individual homopolymers. For block copolymer of the highest PTMC content the band at 1242 cm^−1^ dominates and the ether band of PDLA cannot be discerned. Due to the overlapping of the carbonyl bands in both homopolymers, in block copolymers we observe only one signal at 1744–1748 cm^−1^. Also, the spectra of random copolymers look similar, with two discernible ether bands for 59% PTMC and only one visible for 81% PTMC, and a single carbonyl band at 1742–1750 cm^−1^.

**Figure 2:**
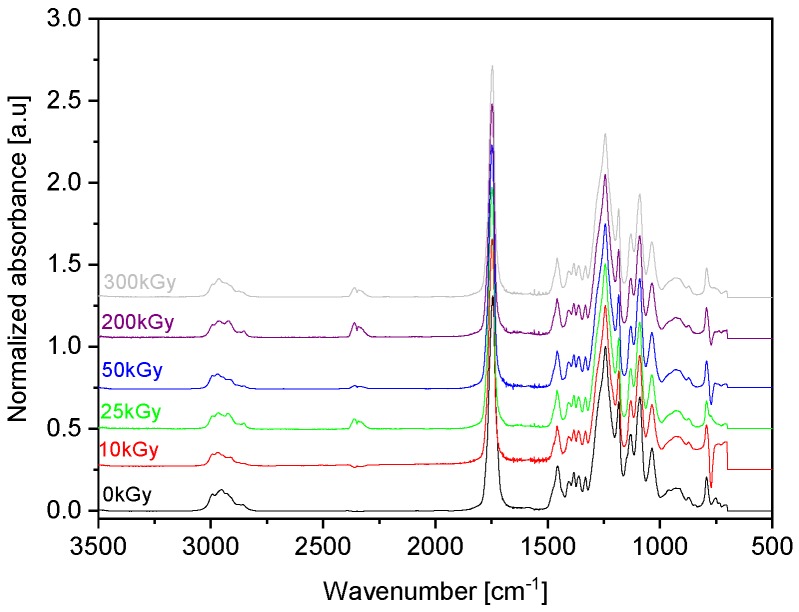
FT-IR spectra of poly(TMC-*rand*-DLA) (59/41) copolymers after irradiation at 0–300 kGy.

Figure 1 shows the FT-IR spectra of the irradiated copolymers and polymers at dose level of 100 kGy. Figure 2, however, shows the FT-IR spectra of the irradiated poly(TMC-*rand*-DLA) (41/59) copolymer with e-beam, at dose levels of 10, 25, 50, 200, and 300 kGy. Additionally, comparison of spectra recorded for the initial sample and samples subjected to EB irradiation up to 300 kGy is presented in Figure 2 for an example of the random copolymer containing 59% PTMC. The FT-IR spectra of all irradiated and un-irradiated poly(TMC-*b*-DLA) and poly(TMC-*rand*-DLA) copolymers, and PTMC, PDLA polymers exhibit similar absorption bands up to 300 kGy, demonstrating that irradiation does not significantly alter their chemical structures.

**Figure 3:**
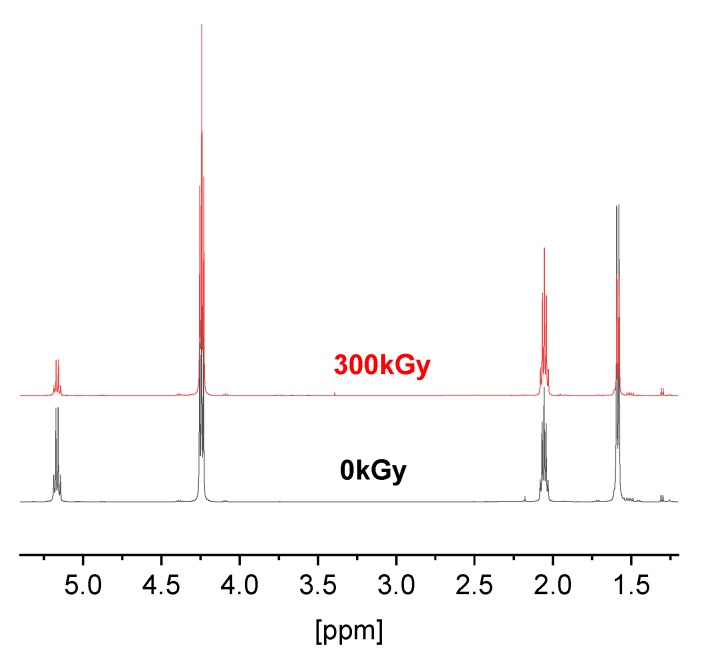
^1^H NMR spectra of the poly(TMC-*b*-DLA) 59/41 diblock copolymer, unirradiated and irradiated with a dose of 300 kGy.

^1^H NMR spectrum of the exemplary block copolymer poly(TMC-*b*-DLA) 59/41 is shown in Figure 3. The spectrum is characterized by typical signals of PDLA main chain methine protons at 5.12 ppm (–CH–), main chain methyl protons at 1.6 ppm (–CH_3_), PTMC main chain methylene protons adjacent to the carbonate group at δ 4.22 (–CH_2_–CO–) and PTMC main chain methylene protons at δ = 2.03 (quintet, –CH_2_–). The minor but well-defined signal at about 1.29 ppm corresponds to the tail-end-initiator residue (-CH(CH_3_)_2_). Figure 3 shows the ^1^H NMR spectra for irradiated poly(TMC-*b*-DLA) 59/41 with 300 kGy. The NMR spectra of all copolymers irradiated and un-irradiated exhibit similar bands (data not presented here). Similarly, to the FT-IR results, the ^1^H NMR results also demonstrated that there are no significant changes in the chemical structures of the irradiated and un-irradiated homo- and copolymers. Irradiation with a sterilization dose (25 kGy) does not influence the structure of the polymers to an extent which would compromise their chemistry.

### 3.3. Intrinsic Viscosity

**Figure 4:**
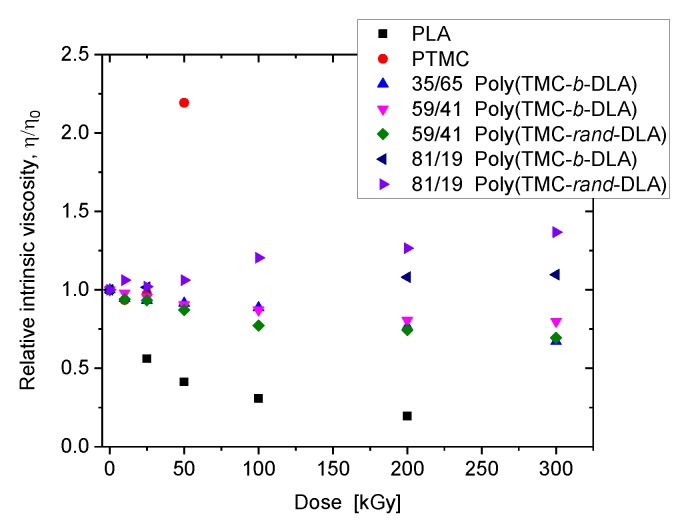
Relative change of the intrinsic viscosity (η/η_0_ — intrinsic viscosities, after irradiation to initial) of diblock and random copolymers compared to those of homopolymers as a function of absorbed dose.

In this work intrinsic viscosity measurements were carried out to qualitatively investigate the presence and the absence of the crosslinking and degradation reactions in the homo- and copolymers as a function of dose. Figure 4 shows PTMC’s [η] increases sharply with increasing dose. These data demonstrate that in the case of homopolymers, PTMC undergo mainly crosslinking reactions. On the other hand, radiation induces degradation in PDLA. In the case of copolymers, however, crosslinking and scissions take place as a function of their compositions. Figure 4 also shows that the copolymers which contain 36% and 59% poly(TMC-*b*-DLA) undergo degradation. On the contrary, 81% poly(TMC-*rand*-DLA) undergoes crosslinking as evidenced by the increase in the [η] Slight increase in [η] can also be detected in the 81% poly(TMC-*b*-DLA).

### 3.4. Molecular Weight

The initial molecular weights of the synthesized copolymers were relatively low, in the order of a few tens of kDa, as compared to the homopolymers. It should be mentioned, that the main radiation-induced effects in polymers irradiated in solid state are not dependent on the molecular weight, except for short oligomers where end-groups comprise significant part of the main chain [34]. Table 2, Table 3 and Table 4 show number- and weight-average molecular weights of unirradiated and irradiated homopolymers and copolymers.

As demonstrated earlier by viscosity measurements, radiation-induced degradation is predominant for PDLA, while crosslinking reaction mainly takes place in PTMC. Copolymers of high PDLA content poly(TMC-*b*-DLA) (35/65), poly(TMC-*b*-DLA) (59/41) and poly(TMC-*rand*-DLA) (59/41)) undergo degradation. These results show that molecular weight dispersion (*M*_w_/*M*_n_) increases in all cases. While for the random copolymer it does not exceed 2, it becomes higher than 2 for the block copolymers. Should only random scissions take place in these samples, one would expect that (*M*_w_/*M*_n_) would approach value of 2 [49], but not to exceed this value. The observed increase of *M*_w_/*M*_n_ higher than 2 indicates that scissions are accompanied by less probable crosslinking reactions, which leads to significant broadening of molecular weight distribution. Since *M*_w_/*M*_n_ does not exceed 2 in the dose range of 10–300 kGy, it is still not clear, if poly(TMC-*rand*-DLA) (59/41) scission and crosslinking occur simultaneously.

An increase in the average molecular weight is observed for both copolymers of the highest PTMC content — poly(TMC-*b*-DLA) (81/19) and poly(TMC-*rand*-DLA) (81/19). Similar to the viscosity results, a relative increase in molecular weights is higher for the random copolymer.

Attention should be paid to the effects of irradiation with the dose of 25 kGy which is regarded as typical sterilization dose. While it is evident that application of radiation as a sterilization method of biomaterials and medical devices comprising pure polylactides or copolymers containing a majority of polylactides is rather unfeasible, it is demonstrated that poly(TMC-*co*-DLA)copolymers of high PTMC content do not degrade or even can be crosslinked by electron beam when irradiated with 25 kGy and higher doses. In irradiated copolymers intermolecular crosslinking and chain scission occur simultaneously, however the crosslinking of PTMC segments or monomer units dominates at high PTMC content. The observed effect of increase in molecular weight for PTMC-rich copolymer resembles, but it is not that pronounced, that occurring in PTMC homopolymer. In the case of neat PTMC an increase in molecular weight eventually leads to formation of insoluble fraction. Macromolecules become chemically crosslinked to form stable gel, which in turn enhances mechanical properties of materials based on PTMC [37]. It is anticipated that lactide biomaterials can withstand radiation sterilization when combined with crosslinking-type co-component. As compared to blending of two polymers, copolymerization seems to be better solution to obtain mixture at molecular level.

**Figure 5:**
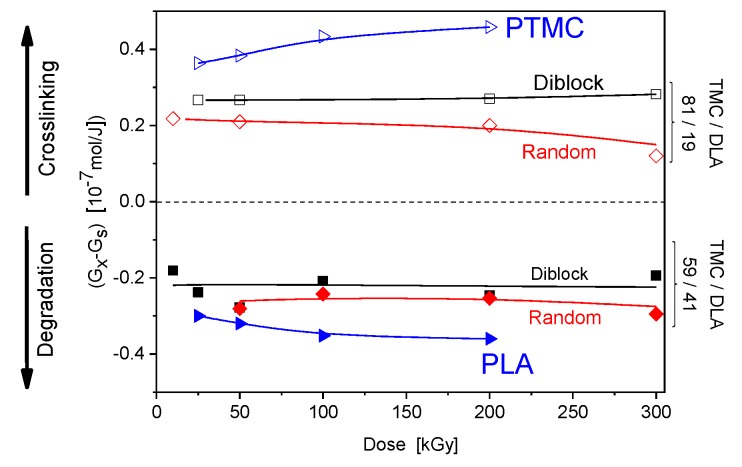
Difference radiation yields of crosslinking and scission (*G*_x_-*G*_s_) for homo- and co-polymers of PTMC and PDLA as a function of dose.

To characterize crosslinking and scission processes occurring in the irradiated polymers, the respective radiation-chemical yields were determined. These important parameters of radiation sensitivity of a particular polymer, radiation chemical yields of chain scission *G*_s_ and intermolecular cross-linking *G*_x_, are defined as the number of moles of scission events or formed crosslinks per unit of absorbed energy. In the polymeric systems where chain scission and crosslinking take place simultaneously, radiation yields can be calculated from the following equations [50]

(2) $$\frac{1}{M_{w}}=\frac{1}{M_{w0}}+\left(\frac{G_{s}}{2}-2G_{x}\right)\cdot D$$

(3) $$\frac{1}{M_{n}}=\frac{1}{M_{n0}}+\left(G_{s}-G_{x}\right)\cdot D$$

where: *M*_w0_ and *M*_n0_ are the initial weight- and number-average molecular weights of the polymer, *M*_w_ and *M*_n_ are the weight- and number-average molecular weights of irradiated samples (all in kg/mol), D is the absorbed dose, *G_x_* is the yield of crosslinking, *G*_s_ is the yield of chain scission (both in mol·J^−1^). While Equation (3) can be used for any molecular-weight distribution of the initial samples, Equation (2) is strictly true only if *M*_w0_/*M*_n0_ = 2. Since this condition is not fulfilled for majority of our samples, we limit the analysis to calculating the value of *G*_x_-*G*_s_, which can be taken as an indication whether in the polymeric system subjected to irradiation crosslinking predominates over scission. The results for homopolymers and poly(TMC-*co*-DLA) copolymers are illustrated in Figure 5. The dominance of crosslinking over chain scission reactions in irradiated samples of copolymers containing higher content of the carbonate blocks is ascertained. The average *G*_x_-*G*_s_ values, the difference of radiation yields of cross-linking and scission, for PTMC-rich (81 mol %) copolymers were calculated to be 0.27 × 10^7^ mol·J^−1^ and 0.22 × 10^−7^ mol·J^−1^, respectively, for diblock and random copolymers, whereas for the PTMC homopolymer the yields difference was over 0.35 × 10^−7^ mol·J^−1^ within investigated absorbed dose range. The average *G*_x_-*G*_s_ values for copolymers with lower content of PTMC, 59 mol %, are negative, and were determined to be −0.21 × 10^−7^ mol·J^−1^ and −0.26 × 10^−7^ mol·J^−1^ for diblock and random copolymers, respectively. Naturally, they are still somewhat higher than that obtained for neat PLA, −0.32 × 10^−7^ mol·J^−1^, since crosslinking is minor for poly(lactic acid).

### 3.5. Thermal Properties

**Figure 6:**
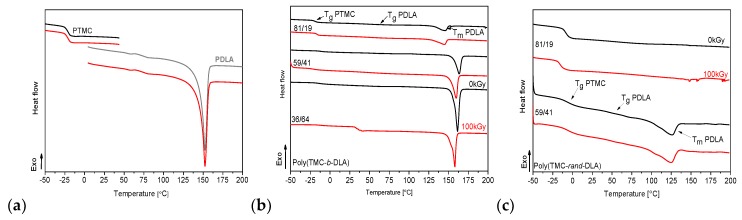
DSC first heating thermograms of homopolymers (**a**), diblock copolymers (**b**) and random copolymers (**c**) with different content of comonomers TMC/DLA.

**Figure 7:**
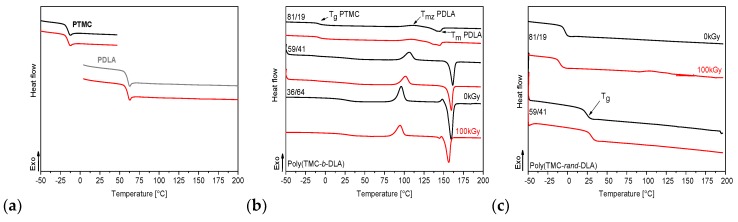
DSC second heating thermograms of homopolymers (**a**), diblock copolymers (**b**) and random copolymers (**c**) with different content of comonomers TMC/DLA.

DSC heating thermograms of neat PDLA and PTMC and their copolymers with different content of monomers are shown in Figure 6, the first heating, and Figure 7 the second heating, respectively. Two heat effects at temperatures corresponding to the glass transition temperature (*T*_g_) and melting temperature (*T*_m_) can be observed the PDLA and block copolymers. PTMC homopolymer and random copolymer of 81/19 are amorphous, whereas that of 59/41 molar composition displays some crystalline phase. Depending on the composition, the glass transition temperatures of the copolymers vary between the *T*_g_ of PTMC (approximately −21 °C) [41] and the *T*_g_ of PDLA (approximately 61 °C) [51]. Glass transition of both blocks is observed in the first heating thermograms at temperatures of ca. −19 ÷ −13 °C for PTMC segments and at temperatures of 45–50 °C for PDLA segments, (Figure 6 black line). The *T*_g_ of both segments slightly increased with an increase in lactide content. In random copolymer of the molar percentage ratio poly(TMC-*rand*-DLA) 59/41 two glass transitions at −6.3 and 44.5 °C were also detected, but for poly(TMC-*rand*-DLA) (81/19) only a single glass transition was observed at −8.8 °C. Lower randomness of 59/41 copolymer (0.19; in the scale where 0 is block and 1 is fully random copolymer) resulted in occurrence of two glass transitions representing separated phases of PTMC and PDLA segments [44]. The randomness of the copolymer chain has a value of 0 in the case of block copolymer and 1 in the case of a completely random distribution of the copolymer repeating units.

Concerning the second heating the only one *T*_g_ transition for all the copolymers (Figure 7) was detected. It relates to stresses generated due to difference in thermal expansion of both constituents. The first heating thermograms for the diblock and random copolymers shows only one endothermal transition of melting (at *T*_m_), whereas second heating of the diblock copolymer thermogram shows, along with melting, additional cold crystallization (at *T*_c_). Cold crystallization is an exothermic crystallization process. It is observed on heating a sample that has previously been cooled very quickly and has had no time to crystallize. Below the glass transition, molecular mobility is severely restricted and cold crystallization does not occur; above the glass transition, small crystallites are formed at relatively low temperatures. Depending of the composition of copolymers a heat transfer resulting from melting of the PDLA indicates its crystalline phase. Detailed comparison of thermal parameters for copolymers of PTMC and PDLA was presented in our previous work [44].

Thermograms of the irradiated diblock copolymers (red lines in Figure 6 and Figure 7 for first and second heating, respectively) show some difference in shapes of the peaks related to their melting temperatures. However, there is no alternation of the shapes of the random copolymers thermograms.

**Figure 8:**
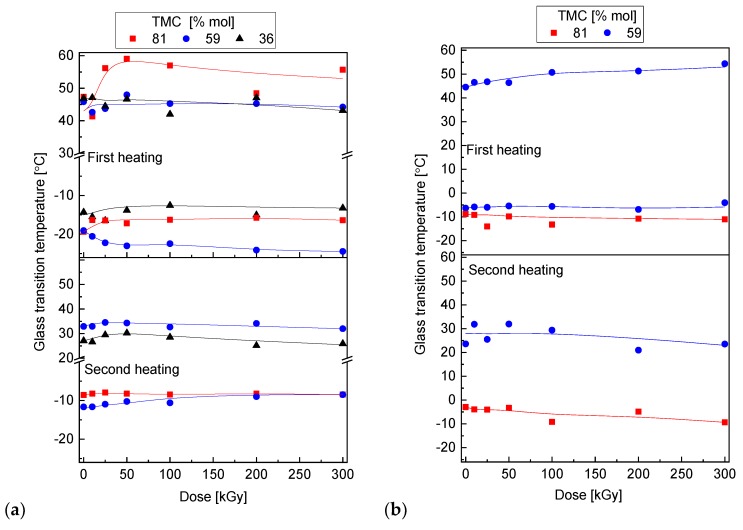
Glass transition temperature of PDLA and PTMC as a function of radiation dose; (**a**) diblock and (**b**) random copolymers.

The data in Figure 8 show the glass transition temperature (*T*_g_) of diblock and random copolymers of various compositions irradiated to doses of up to 300 kGy. A general observation is that after irradiation all glass transitions exist at very similar level as for non-irradiated samples. *T*_g_ of TMC component in either block and random copolymers does not change after irradiation. Even if the transition point of the non-irradiated random copolymers is shifted towards higher temperatures due to presence of the co-component segments, it remains at the same level. The observation is consistent with the literature data for neat PTMC for which *T*_g_ is constant through wide range of absorbed doses [37]. Degradation of lactide blocks or segments however, does not result in reduction of glass transition temperature of the PDLA component, as could be expected. It was reported that extensive degradation of the PLA is reflected in the decrease in the *T*_g_ [40]. This may happen if ability of segmental motion is altered due to extensive degradation — short chains formation and creation of end-groups in large number, thus disruption of polymer chemistry. Apparently, in the present case the degradation of already relatively short lactide chains does not change the lability of segments. This is partially related to presence of the carbonate component that absorbs a part of energy which is proportional to its weight fraction.

**Figure 9:**
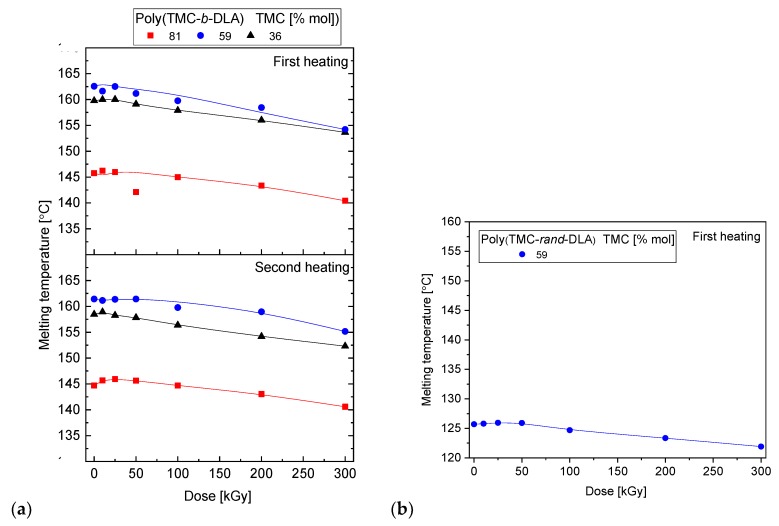
Melting temperature of diblock (**a**) and random (**b**) copolymers as a function of absorbed dose.

PDLA is an intrinsically semicrystalline polymer which exhibits a melting peak at 170–195 °C [52] depending on its isomeric purity, thus it was expected that its block copolymers would be also semicrystalline. A continuous decrease in melting temperature of PLA segments with respect to increasing radiation dose is due to polylactide degradation. The effect is strong and visible in the first and second heating scans (Figure 9). It is preceded by the cold crystallization in the latter case (longer chains of lactide blocks crystallize at lower temperature — lower temperature (lesser energy) is required to re-orientate the shorter chains). Shorter chains possess better movement ability, therefore partially degraded PLA can adjust into ordered configuration earlier, that is at lower temperature while heating over the *T*_g_. Even over 5 °C decrease in the *T*_m_ was recorded for samples irradiated with a relatively high dose of 300 kGy. However, up to ca. 50 kGy the *T*_m_ remains unchanged, showing that a specific quantity of chains which are degraded is required to result in *T*_m_ decrease. One may expect that plastification effect of newly created shorter chains plays its role, even though it is not demonstrated in the alternation of the *T*_g_. Random copolymer of the higher lactide content (59/41 TMC/DLA) also exhibited presence of regular segmental arrangement, with relatively low Tm of ca. 126 °C which was further decreasing of about 4 degrees after irradiation with the highest applied dose.

**Figure 10:**
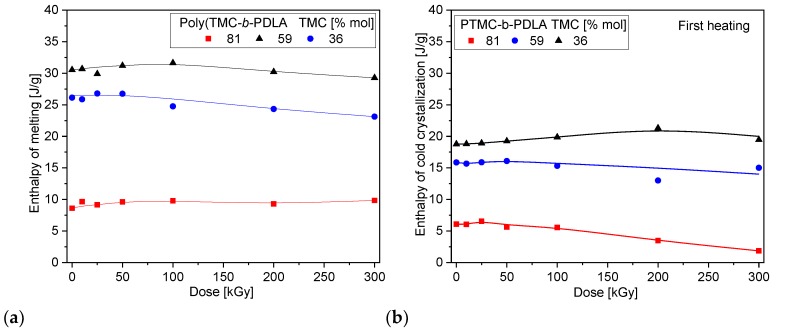
Heat of melting (**a**) and cold crystallization (**b**) of diblock as a function of absorbed dose.

The crystallization ability of these DLA-based copolymers is affected by DLA content and its average sequence length; however, this does not change with dose as identified in the first heating. Figure 10 shows heat of fusion related to melting of crystalline phase of the lactide, which in the case of block copolymers is preceded by cold crystallization. Exothermic peak of cold crystallization was not observed for random copolymers of 59/41 TMC/DLA. Surprisingly, the degree of crystallinity, as represented only by the DLA fraction, does not change significantly with even high absorbed dose. It is known that poly(lactic acid) slightly increases its degree of crystallinity upon irradiation [30]. Degradation usually facilitates crystallization to larger extent because of easier organization of shorter chains. In the copolymer with flexible segments as provided by TMC, crystallization of DLA part is intrinsically high — of ca. 50–65%, thus appearance of shorter chains does not further increase the *χ*_c_.

**Figure 11:**
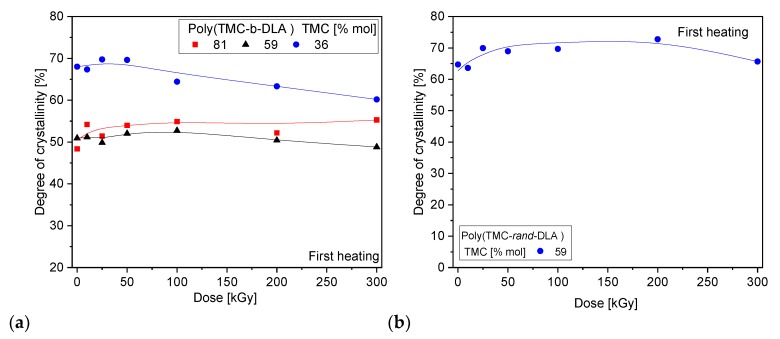
Degree of crystallinity of diblock (**a**) and random (**b**) copolymers as a function of absorbed dose.

For diblock copolymers with 81% TMC a slight increase in the degree of crystallinity was detected during first heating (Figure 11). The initial increase in degree of crystallinity is due to the re-orientation of the shorter chains of PDLA. In the case of a diblock copolymer with 59% PTMC it was found that the degree of crystallinity does not change at initial radiation doses, then over 50 kGy a slight decrease was observed.

The DSC measurements demonstrated that, even while degradation and crosslinking occur in the copolymers, the overall thermal properties are controlled rather by the molar composition and microstructure of copolymers but not by the irradiation.

### 3.6. Radiolytically Produced Free Radicals

**Figure 12:**
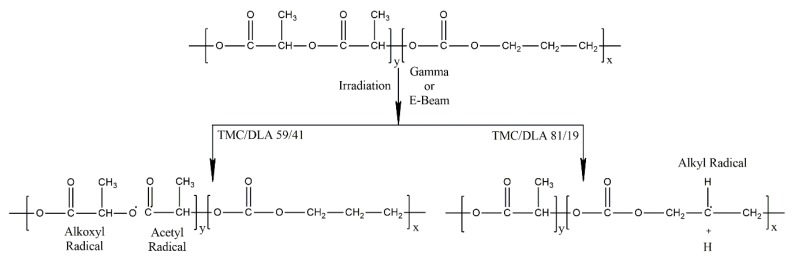
Formation of alkoxy and carbon center radicals upon irradiation of poly(TMC-*b*-DLA).

Based on the chemical structure of the poly(TMC-*b*-DLA), it is expected that ionizing radiation induces ester bonds scission giving rise to alkoxyl and acetyl radicals, and simultaneously the alkyl radicals are formed in the PTMC segments, as shown below (Figure 12).

As demonstrated in Figure 12, while the probability of the ester bonds scission is proportional to the DLA component with the production of alkoxyl and alkyl radicals, the cleavage of C–H bonds along the backbone of the chains becomes more prevailing as the TMC component increases.

**Figure 13:**
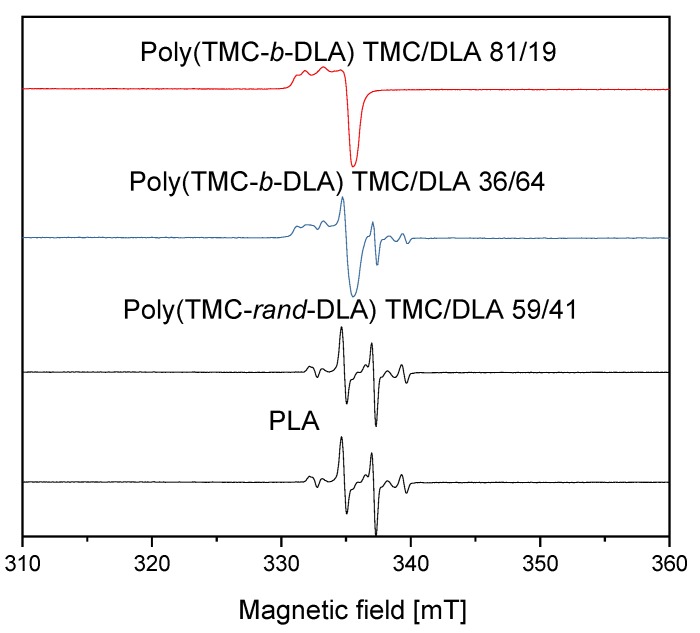
EPR spectra of diblock and random copolymers of various compositions irradiated by EB to dose of 30 kGy at dry ice temperature and recorded at room temperature immediately after irradiation. Spectrum of PLA radicals is shown for comparison.

**Figure 14:**
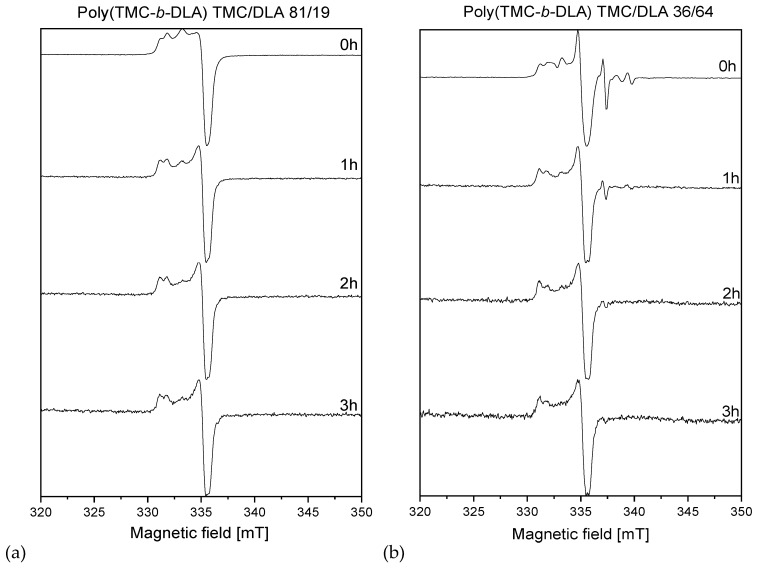
EPR spectra of diblock copolymers poly(TMC-*b*-DLA) (**a**) TMC/DLA 81/19 (**b**) TMC/DLA 36/64 irradiated by EB to dose of 30 kGy at dry ice temperature.

The EPR spectra of the irradiated diblock and random copolymers are presented in Figure 13 together with the spectrum obtained for PLA homopolymer. In every case, these samples were irradiated with electron beam at total dose of 30 kGy, in the absence of oxygen and in the presence of dry ice (−78.5 °C). PTMC homopolymer displayed no measurable spectra, since at room temperature radical created upon irradiation decayed immediately in this amorphous polymer. The time evolution of the spectra of the irradiated diblock copolymers poly(TMC-*b*-DLA) with TMC/DLA 36/64 and TMC/DLA 81/19 are shown in Figure 14 respectively. The changes of the spectra were monitored in the absence of oxygen and at room temperature. It is clear that the EPR spectra of TMC/DLA 36/64 and TMC/DLA 81/19, which were measured immediately after irradiation, are different. For TMC/DLA 36/64, where the formation of the alkoxyl is relatively high as a result of the cleavage of the ether bonds in the lactide, the EPR spectra of the irradiated samples exhibit spectrum very similar to the alkoxyl radical [53]. The hyperfine splitting of the EPR spectrum can be calculated by the following general formula: (2M_1_I_1_ + 1) = number of the signals, where M is the number of the adjacent H atoms, and I is spin 1 = ½ [54].

**Figure 15:**
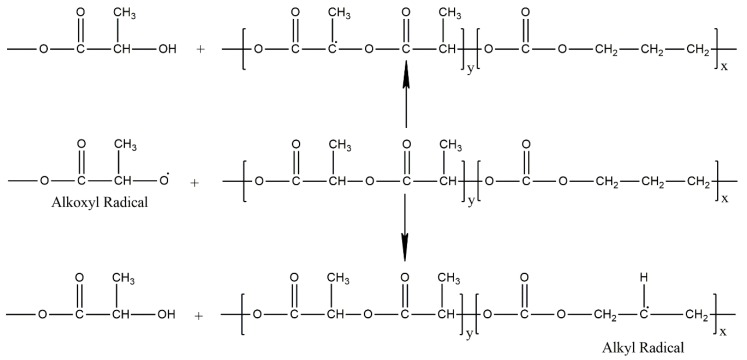
The abstraction of the H-atom from neighboring diblock copolymer poly(TMC-*b*-DLA) chains by the alkoxyl radical.

As expected, the EPR spectrum of the irradiated block copolymers TMC/DLA 36/64, exhibit singlet, which attributes to the acetyl radical, and a doublet, which can be assigned to the alkoxyl radical. It is well known that, similar to the peroxyl radical, alkoxyl radical EPR signals are relatively week, and very broad. Other contributing factor resides in the fact that alkoxyl radicals undergo relative fast decay via abstraction of H-atoms from the neighboring chains producing hydroxide and alkyl radicals as follows, as presented in Figure 15.

The EPR spectra of the irradiated TMC/DLA 81/19 are shown in Figure 14. Since the TMC content is very high, as mentioned, it is expected that the EPR spectrum present sextet for the alkyl radical in the TMC segment, similar to the free radical in polyethylene, in addition to the contribution from alkoxyl radicals. However, the spectrum, which was measured immediately after irradiation, exhibits a weak quadruplet, in addition to the singlet of the acetyl, and very weak doublet of the alkoxyl radical. The exhibit of the weak quadruplet rather than sextet may be contributed to the fast transformation of the TMC-located alkyl to allyl radicals [55,56]. Another contribution to the quadruplet may be the tertiary alkyl radical located at the PLA segment (which is known to be formed in PLA homopolymer [55]). The weakness of the quadruplet may also contribute to the fact that alkyl radical undergoes bimolecular crosslinking — as explained earlier, and disproportionation reactions. As also shown in Figure 15 the evidence of the crosslinking and disproportionation can be seen in the relatively fast disappearance of the quadruplet.

It should also be mentioned that, as expected, the singlet of the acetyl radical stays relatively stable in comparison with the other free radicals. The relative stability of acetyl radical can clearly be explained by the presence of conjugated system. The presence of the carbonyl system C=O on the same C atom of the free radical provides an excellent conjugated system that decreases the density of the negativity leading to less reactive free radicals. However, the main factor of the stability of the acetyl and alkoxyl radicals is that they are in the DLA component, which is the crystal part of the TMC-DLA copolymer.

**Figure 16:**
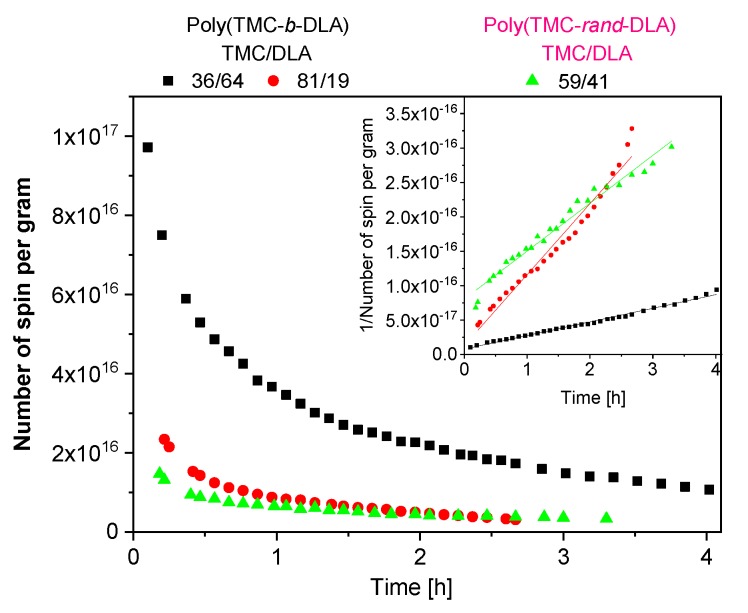
Free radical concentration and decay fitted to second order kinetics (inset) of diblock and random copolymers irradiated by EB to a total dose of 30 kGy at dry ice temperature as measured at room temperature. Irradiation and measurement without under protective gas.

Figure 16 shows the effects of the copolymers composition and the microstructure the on the immediate free radical concentration. As the DLA/TMC ratio increases, the immediate free radical concentration, in number of spin per gram, increases. This is clearly related to the content of ester linkages, since DLA contains in its chemical structure more ester groups than TMC, and to the increase in crystalline fraction.

The overall decays of theses free radicals were monitored as a function of time and the DLA/TMC ratios. The inset in Figure 16 shows the second order decay of these radiolytically produced free radical — the predominant second order decay fits. It is very well accepted that in the absence of oxygen, the radiolytically produced alkyl radicals undergo second order crosslinking reactions [55]. It should also be mentioned that these observed second order decays may very well include pseudo-first order component from the abstraction of H atoms along the backbone of the chain by the alkoxyl radicals leading to the formation of alkyl radicals and hydroxide. This may explain the reason for some deviations from the straight lines of these second order fits (Figure 16).

In the random copolymer a spectrum resembling that for PLA homopolymer was recorded (Figure 13). Since the random copolymer forms crystalline phase (as explained earlier [44]) it can contain trapped radicals at room temperature. Nevertheless, the number of radicals is relatively low (despite that the DLA mass content is 42%), lower than for block copolymer with only 19% DLA. Obviously the most stable radicals remain, and they decay slower than those in the block copolymer (Figure 16). Second order kinetics is predominant, but there may be still a fraction of faster decaying radicals since there is some initial deviation from the perfect second order fit. Those stable radicals may be diverse. H abstraction from methine groups located in the PLA units of the polymer chain creates tertiary alkyl radicals •C(CH_3_)– (stable quartet) that, as in PLA homopolymer, decay slowly under vacuum because the carbonyl function assists in stabilization of the fragment [57]. Some relatively well stabilized allyl radicals may also be formed in the TMC units (see above) and contribute to the quartet signal. Alkoxyl and acetyl radicals, as the result of ester linkages scission, same as in block copolymers, are present as well. This corresponds well with the thermal characteristics of the irradiated samples, i.e., melting temperature of lactide crystallites was considerably decreasing with irradiation (Figure 9) that evidenced degradation of the DLA component.

## 4. Conclusions

The results of this study demonstrate that the radiation chemistry of the trimethylene-lactide copolymers strongly depends on the presence of ester linkages, and therefore on the TMC/DLA ratio of this copolymer. It is concluded from the results of all techniques used in this study that as the ester group content increases, the probability of copolymers degradation increases. Only in copolymers with lowest ester linkage number, i.e., at the highest TMC content, the yield of crosslinking surpasses the yield of scission, likewise in the carbonate homopolymers.

EPR studies on irradiated poly(TMC-*b*-DLA) block and random copolymers indicate that the presence of amorphous polycarbonate units leads to the decrease of the radiolytic yield of radicals. The complex EPR spectra show the production of alkoxyl and acetyl radicals because of ester linkages scission. Because ester linkages scission occurs in mostly crystalline region, since DLA forms ordered structure, as demonstrated by DSC experiments, one would expect the remarkable stability of both alkoxyl and the acetyl radicals. In addition, the conjugated system in the acetyl radical enhances the stability of this radical. Moreover, the production of alkyl radicals at TMC segments were expected, which becomes the dominant reaction at high TMC content. However, the EPR spectra did not show a sextet of the alkyl radical as expected. Instead, the EPR spectra show weak quadruplet suggesting the formation of the allyl radicals in the TMC segments and tertiary alkyl radicals in the PLA units. Therefore, one can conclude that the initially formed secondary alkyl radicals in TMC fragments undergo relatively fast decay, e.g., recombination or crosslinking reactions and unimolecular transformation to allyl radicals.

Radiation causes essentially no significant changes in the functional groups of the copolymers, especially at a relatively low dose as typically applied for sterilization. Even though the chemical composition is not altered, irradiation induces pronounced changes in the properties of the poly(TMC-*co*-DLA) copolymers due to the increase or decrease of molecular weight, depending on the monomer ratio. Practical implications may be that, through copolymerizing the lactide with TMC segments, it is possible not only to modify the properties of rigid PLA, but also to introduce certain resistance of this radiation-degradable polymer against destructive action of ionizing radiation.

## Figures and Tables

**Table 1:** Characteristics of trimethylene carbonate (TMC) and d-lactide (DLA) copolymers used in the study.

Type of Copolymer	TMC Fraction [mol %]		Conv. [%]	*L*_DLA_^a^	*L*_TMC_^a^	*M*_n_	*M*_w_	*M*_w_/*M*_n_
	Feed	Actual						
Poly(TMC-*b*-DLA)	36	36	98	-	-	19	31	1.63
Poly(TMC-*b*-DLA)	59	59	99	-	-	24	47	1.96
Poly(TMC-*b*-DLA)	81	81	99	-	-	18	35	1.94
Poly(TMC-*rand*-DLA)	59	58	100	13.0	6.6	18	20	1.11
Poly(TMC-*rand*-DLA)	81	81	100	3.8	14.0	17	19	1.12

^a^ Average length of the LA and TMC microblocks calculated with ^13^C NMR [44].

**Table 2:** Number-average and weight-average molecular weights [kg·mol^−1^] and molecular weight dispersion of PTMC and PDLA homopolymers as a function of dose.

Dose [kGy]	PTMC			PLA		
	*M*_n_	*M*_w_	*M*_w_/*M*_n_	*M*_n_	*M*_w_	*M*_w_/*M*_n_
0	108.9	270	2.48	215	260	1.21
10	124.4	260	2.09	-	-	-
25	136.5	277	2.03	90	105	1.17
50	142.6	290	2.03	62	96	1.55
100	162.3	308	1.90	45	70	1.56
200	202.5	336	1.66	30	68	2.27

**Table 3:** Number-average and weight-average molecular weights [kg·mol^−1^] and molecular weight dispersion of poly(TMC-*b*-DLA) diblock copolymers of various composition (in mol %).

Dose [kGy]	Poly(TMC-*b*-DLA)								
	81/19			59/41			36/64		
	*M*_n_	*M*_w_	*M*_w_/*M*_n_	*M*_n_	*M*_w_	*M*_w_/*M*_n_	*M*_n_	*M*_w_	*M*_w_/*M*_n_
0	19	31	1.63	24	47	1.96	18	35	1.94
10	-	-	-	23	45	1.96	17	32	1.88
25	30	56	1.87	21	44	2.10	15	31	2.07
50	28	43	1.54	18	37	2.06	14	30	2.14
100	29	41	1.41	16	39	2.44	12	27	2.25
200	50	78	1.56	13	29	2.23	9	19	2.11
300	65	82	1.26	10	23	2.30	7	19	2.71

**Table 4:** Number-average and weight-average molecular weights [kg·mol^−1^] and molecular weight dispersion of poly(TMC-*rand*-DLA) random copolymers, of various composition (in mol %).

Dose [kGy]	Poly(TMC-*rand*-DLA)					
	81/19			59/41		
	*M*_n_	*M*_w_	*M*_w_/*M*_n_	*M*_n_	*M*_w_	*M*_w_/*M*_n_
0	18	20	1.11	17	19	1.12
10	19	21	1.11	14	16	1.14
25	-	-	-	12	15	1.25
50	28	30	1.07	12	15	1.25
100	31	35	1.13	10	13	1.30
200	39	46	1.18	7	10	1.43
300	43	49	1.14	6	9	1.50
